# Dynamic BOLD functional connectivity in humans and its electrophysiological correlates

**DOI:** 10.3389/fnhum.2012.00339

**Published:** 2012-12-28

**Authors:** Enzo Tagliazucchi, Frederic von Wegner, Astrid Morzelewski, Verena Brodbeck, Helmut Laufs

**Affiliations:** Neurology Department and Brain Imaging Center, Goethe UniversityFrankfurt am Main, Germany

**Keywords:** dynamic connectivity, EEG-fMRI, resting state, brain networks, brain oscillations

## Abstract

Neural oscillations subserve many human perceptual and cognitive operations. Accordingly, brain functional connectivity is not static in time, but fluctuates dynamically following the synchronization and desynchronization of neural populations. This dynamic functional connectivity has recently been demonstrated in spontaneous fluctuations of the Blood Oxygen Level-Dependent (BOLD) signal, measured with functional Magnetic Resonance Imaging (fMRI). We analyzed temporal fluctuations in BOLD connectivity and their electrophysiological correlates, by means of long (≈50 min) joint electroencephalographic (EEG) and fMRI recordings obtained from two populations: 15 awake subjects and 13 subjects undergoing vigilance transitions. We identified positive and negative correlations between EEG spectral power (extracted from electrodes covering different scalp regions) and fMRI BOLD connectivity in a network of 90 cortical and subcortical regions (with millimeter spatial resolution). In particular, increased alpha (8–12 Hz) and beta (15–30 Hz) power were related to decreased functional connectivity, whereas gamma (30–60 Hz) power correlated positively with BOLD connectivity between specific brain regions. These patterns were altered for subjects undergoing vigilance changes, with slower oscillations being correlated with functional connectivity increases. Dynamic BOLD functional connectivity was reflected in the fluctuations of graph theoretical indices of network structure, with changes in frontal and central alpha power correlating with average path length. Our results strongly suggest that fluctuations of BOLD functional connectivity have a neurophysiological origin. Positive correlations with gamma can be interpreted as facilitating increased BOLD connectivity needed to integrate brain regions for cognitive performance. Negative correlations with alpha suggest a temporary functional weakening of local and long-range connectivity, associated with an idling state.

## 1. Introduction

Neural oscillations at specific frequency bands reflect a wide repertoire of brain states, ranging from active cognitive performance to idling rest, sleep and other states of diminished awareness (Buzsáki, 2006). Experimental results relate power increases and synchronization in the gamma frequency band (≈40 Hz) to the performance of perceptual and cognitive operations, including attention (Womelsdorf and Fries, 2007), conscious perception (Melloni et al., 2007), and decision making (Donner et al., 2009). In particular, the ubiquity of gamma band synchronization in human brain function has led to its postulation as a fundamental process subserving an elementary operation of cortical computation (Fries, 2009). When compared to other alternatives, like rate encoding, neural integration by temporal synchronization offers many theoretical advantages, such as, a faster temporal realization and no ambiguities due to modulation of firing rate by other causes (e.g., receptive field tuning or other concurrently integrated neural groups) (Gray et al., 1989). On the other hand, increased power in slower frequencies has been linked with an idling state of the brain (Pfurtscheller et al., 1996) and with functional inhibition of task-irrelevant regions, facilitating routing of information to task-relevant regions (Klimesch et al., 2007; Jensen and Mazaheri, 2010). Slower frequencies, such as delta (<4 Hz), arise during sleep and are hypothesized to reflect diminished temporal complexity underlying loss of conscious awareness (Tononi et al., 1994; Tononi, 2004, 2008).

This integration and inhibition of cortical processes through local and long-range neural synchrony translates into temporal fluctuations of functional connectivity, observable by electrophysiological recordings (such as scalp electroencephalography—EEG). Dynamic functional connectivity has only recently been demonstrated for Blood Oxygen Level-Dependent (BOLD) signals, measured with functional Magnetic Resonance Imaging (fMRI) (Chang and Glover, 2010; Tagliazucchi et al., 2010a,b, 2012; Handwerker et al., 2012; Hutchison et al., 2012; Petridou et al., 2012; Smith et al., 2012). Given the precise spatial resolution and whole brain coverage allowed by fMRI, understanding the relationship between brain rhythms and BOLD connectivity could contribute to the identification of the functional roles of each oscillation at rest and during a task. A necessary first step in this direction is to understand the relationship between dynamic BOLD connectivity fluctuations and changes in the local synchronization of cortical neural populations, as indexed by frequency-specific EEG power. Large efforts have been devoted to the identification of the electrophysiological counterparts of fMRI BOLD signal amplitude during wakeful rest. Along this line, it has been demonstrated that EEG power in the alpha band correlates negatively with spontaneous BOLD amplitude fluctuations in lateral frontal and parietal cortices, whereas power in the beta band correlates positively with regions comprising the Default Mode Network [a network of task de-activated regions (Raichle et al., 2001; Laufs et al., 2003a,b)]. Further evidence for a specific profile of EEG spectral power associated with different Resting State Networks (RSN) was provided in Mantini et al. (2007). In monkey studies, Local Field Potentials (LFP) exhibit widespread positive correlations with BOLD fMRI, mainly in the gamma frequency band (Schölvinck et al., 2010). These reports during rest are complemented by studies mapping the electrophysiological correlates of the BOLD signal during task performance and stimulation, also demonstrating that LFP gamma frequency is a main contributor to the hemodynamic signal (Logothetis et al., 2001; Logothetis and Pfeuffer, 2004). In spite of this large body of studies relating rhythmic neural activity to BOLD signal amplitude, a relationship between BOLD functional connectivity and the power of band-specific scalp oscillations remains a relatively unexplored possibility.

In this paper we study such possibility by correlating dynamic BOLD connectivity fluctuations with changes in EEG power in a group of awake human subjects, as well as in a group of subjects undergoing vigilance transitions between wakefulness and light sleep, whose EEG recordings exhibit marked changes in spectral power over time. The inclusion of these subjects can be seen as a (physiological) manipulation in one of the correlated variables (EEG power in different bands) to test the effect on the other (BOLD functional connectivity). We hypothesize that band-specific electrophysiological spectral changes will correlate with fluctuations in BOLD connectivity and that increased power in fast EEG frequency bands (such as gamma) will correlate with increased BOLD connectivity, considering the ubiquity of gamma oscillations in long-range neural synchronization. On the other hand, activity in the alpha and peri-central (or rolandic) beta bands will not be expected as a contributor to increased BOLD connectivity, given their hypothesized relationship with idling and functional inhibition. We also hypothesize that changes in functional connectivity over time will impact on graph theoretical metrics, which are established descriptors of the global connectivity network architecture. The study of global brain connectivity patterns has been, in recent years, greatly aided by the introduction of graph theoretical concepts (Sporns et al., 2004; Bullmore and Sporns, 2009). These allow to extract information from a network representation of brain functional connectivity, in which nodes represent distinct brain regions and links represent synchronized activity between those regions. The use of these methods not only allows the evaluation of changes in functional connectivity strength, but also in the topological re-organization of brain connectivity and its interpretation in terms of efficient information processing (Bullmore and Sporns, 2009). Finally, an important consequence of our study will be to confirm a neurobiological origin for BOLD connectivity fluctuations, given concerns that they might arise due to motion or physiological confounds (Hutchison et al., 2012) and as artifacts due to sliding window correlation analyses (Handwerker et al., 2012).

## 2. Materials and methods

### 2.1. EEG-fMRI acquisition and artifact correction

EEG via a cap (modified BrainCapMR, Easycap, Herrsching, Germany) was recorded continuously during fMRI acquisition, yielding 1505 volumes of T2^*^-weighted echo planar images with TR/TE = 2080/30 ms, matrix 64 × 64, voxel size 3 × 3 × 2 mm^3^, distance factor 50% and FOV 192 mm^2^. Scanning was performed at 3 T (Siemens Trio, Erlangen, Germany) together with an optimized polysomnographic setting including chin and tibial EMG, ECG, EOG recorded bipolarly (sampling rate 5 kHz, low pass filter 1 kHz), and 30 EEG channels recorded with FCz as the reference (sampling rate 5 kHz, low pass filter 250 Hz). Pulse oxymetry and respiration were recorded via sensors from the Trio (sampling rate 50 Hz) and MR scanner compatible devices (BrainAmp MR+, BrainAmp ExG; Brain Products, Gilching, Germany).

MRI and pulse artifact correction were performed based on the average artifact subtraction (AAS) method (Allen et al., 1998) as implemented in Vision Analyzer2 (Brain Products, Germany) followed by objective (CBC parameters, Vision Analyzer) ICA-based rejection of residual artifact-laden components after AAS, resulting in EEG with a sampling rate of 250 Hz. EEG was re-referenced to common average. Sleep stages were scored manually by an expert according to the AASM criteria (AASM, 2007).

### 2.2. Subjects and datasets

A total of 15 awake subjects were included in the study (10 female, age 26.2 ± 6), together with an independent group of 13 subjects undergoing vigilance transitions between wakefulness and light sleep or N1 sleep (8 female, age 23.3 ± 3.4). In all cases, written informed consent and approval by the local ethics committee were obtained. Both groups were extracted from a larger dataset with the following inclusion criteria: for the first group, subjects did not show any period of sleep (as determined by AASM sleep scoring criteria). For the second group, subjects showed only epochs of wakefulness and at least 20% of light (N1) sleep (the total time spent in N1 sleep was 13.09 ± 6.26 min). According to AASM criteria, an epoch of N1 sleep was scored whenever alpha waves were replaced by low-amplitude and lower frequency (4–7 Hz) waves occupying >50% of the epoch. In the cases in which alpha waves were not visible during wakefulness, the presence of 4–7 Hz oscillations with slowing of background activity (compared with wakefulness), vertex sharp waves, or slow eye movements were also used to score N1 sleep.

### 2.3. fMRI pre-processing

Using Statistical Parametric Mapping (SPM 8, http://www.fil.ion.ucl.ac.uk/spm) EPI data were realigned, normalized (MNI space), and spatially smoothed (Gaussian kernel, 8 mm^3^ full width at half maximum). Cardiac-, respiratory- [both estimated with the RETROICOR method (Glover et al., 2000)] and motion-induced noise were regressed out. fMRI data was bandpass filtered in the range 0.01–0.1 Hz using a 6th order Butterworth filter.

### 2.4. Time dependent correlation matrix

To study the covariance of functional connectivity and EEG power in different frequency bands, an estimate of how the former changes over time is necessary. For this purpose (following, Hutchison et al., 2012; Fraiman and Chialvo, 2012) a sliding window analysis was employed, with a window length of ≈2 min (60 volumes). This window length was chosen because it is relatively short compared to the length of the experiment (≈50 min), while allowing good functional connectivity estimates (Van Dijk et al., 2010). As a first step, the average BOLD signal was extracted from each one of the 90 cortical and subcortical regions defined by the AAL template (Tzourio-Mazoyer et al., 2002) (information on all the regions is provided in Table 1). These time series are notated as *x*_*i*_, 1 ≤ *i* ≤ 90. Next, the time-dependent correlation matrix was obtained as follows:

(1) $$\begin{array}{l} C_{ij}(t)=\frac{\begin{array}{l} \sum_{n=t}^{t+k}\left(x_{i}(n)-\frac{1}{k}\sum_{m=t}^{t+k}x_{i}(m)\right)\times \\ \;\left(x_{j}(n)-\frac{1}{k}\sum_{m=t}^{t+k}x_{j}(m)\right) \end{array}}{\begin{array}{l} \sqrt{\sum_{n=t}^{t+k}{\left(x_{i}(n)-\frac{1}{k}\sum_{m=t}^{t+k}x_{i}(m)\right)}^{2}}\times \\ \sqrt{\sum_{n=t}^{t+k}{\left(x_{j}(n)-\frac{1}{k}\sum_{m=t}^{t+k}x_{j}(m)\right)}^{2}} \end{array}}\\ \;=\frac{\begin{array}{l} \langle (x_{i}(t:t+k)-\langle x_{i}(t:t+k)\rangle )\times \\ (x_{j}(t:t+k)-\langle x_{j}(t:t+k)\rangle )\rangle \end{array}}{\sigma \left(x_{i}(t:t+k)\right)\sigma \left(x_{j}(t:t+k)\right)} \end{array}$$

**Table 1:** **Region number, name, abbreviation, system membership (from Achard et al., 2006), and anatomical coordinates (for the center of mass of the 90 cortical and subcortical regions defined in the AAL template)**.

**# (left–right)**	**Region name**	**Abbreviation**	**System**	**Coordinates (left–right)**
1–2	Precentral gyrus	PCG	Primary	(37, −6, 50) – (−42, −4, 48)
3–4	Superior frontal gyrus	SFG	Association	(17, 34, 41) – (−22, 36, 40)
5–6	Superior frontal gyrus, orbital part	ORBsup	Paralimbic	(13, 48, −17) – (−20, 47, −17)
7–8	Middle frontal gyrus	MFG	Association	(33, 34, 32) – (−36, 34, 32)
9–10	Middle frontal gyrus, orbital	ORBmid	Paralimbic	(28, 53, −14) – (−34, 50, −13)
11–12	Inferior frontal gyrus, opercular	INFoperc	Paralimbic	(46, 17, 19) – (−52, 13, 14)
13–14	Inferior frontal gyrus, triangular	INFtriang	Association	(45, 32, 12) – (−48, 31, 10)
15–16	Inferior frontal gyrus, orbital	ORBinf	Paralimbic	(36, 32, −14) – (−39, 30, −16)
17–18	Rolandic operculum	ROL	Association	(48, −4, 13) – (−50, −8, 11)
19–20	Supplementary motor area	SMA	Association	(4, 3, 60) – (−9, 8, 59)
21–22	Olfactory cortex	Olf	Primary	(5, 16, −14) – (−14, 13, −15)
23–24	Superior frontal gyrus, medial	ORBsupmed	Paralimbic	(4, 52, 28) – (−9, 51, 27)
25–26	Superior frontal gyrus, dorsal	SFGdor	Association	(4, 52, −11) – (−9, 55, −11)
27–28	Rectus gyrus	REC	Paralimbic	(4, 34, −21) – (−9, 37, −22)
29–30	Insula	INS	Paralimbic	(34, 8, 0) – (−38, 7, 0)
31–32	Anterior cingulate gyrus	ACG	Paralimbic	(4, 38, 13) – (−8, 37, 10)
33–34	Middle cingulate gyrus	MCG	Paralimbic	(4, −5, 38) – (−9, −14, 39)
35–36	Posterior cingulate gyrus	PCG	Paralimbic	(4, −40, 19) – (−8, −41, 23)
37–38	Hippocampus	Hip	Limbic	(24, −20, −11) – (−29, −20, −13)
39–40	Parahippocampal gyrus	PHG	Paralimbic	(21, −15, −22) – (−25, −16, −23)
41–42	Amygdala	Amyg	Limbic	(23, 1, −19) – (−27, −1, −20)
43–44	Calcarine cortex	Cal	Primary	(12, −73, 9) – (−11, −79, 5)
45–46	Cuneus	Cun	Association	(10, −79, 28) – (−9, −79, 27)
47–48	Lingual gyrus	Ling	Association	(13, −68, −5) – (−18, −69, −6)
49–50	Superior occipital gyrus	SOG	Association	(20, −78, 31) – (−19, −84, 27)
51–52	Middle occipital gyrus	MOG	Association	(32, −79, 19) – (−35, −80, 15)
53–54	Inferior occipital gyrus	IOG	Association	(33, −82, −7) – (−40, −78, −9)
55–56	Fusiform gyrus	Fus	Association	(29, −40, −21) – (−34, −41, −22)
57–58	Postcentral gyrus	PostCG	Primary	(36, −23, 51) – (−46, −21, 47)
59–60	Superior parietal gyrus	SPG	Association	(22, −56, 61) – (−27, −58, 57)
61–62	Inferior parietal gyrus	IPG	Association	(42, −44, 49) – (−46, −44, 45)
63–64	Supramarginal gyrus	SMG	Association	(52, −29, 33) – (−59, −33, 28)
65–66	Angular gyrus	Ang	Association	(40, −58, 39) – (−47, −59, 33)
67–68	Precuneus	PCUN	Association	(6, −54, 42) – (−10, −54, 46)
69–70	Paracentral lobule	PCL	Association	(3, −29, 66) – (−11, −22, 68)
71–72	Caudate	Cau	Subcortical	(10, 12, 8) – (−15, 11, 7)
73–74	Putamen	Put	Subcortical	(23, 6, 1) – (−27, 4, 0)
75–76	Pallidum	Pal	Subcortical	(16, 0, −1) – (−21, 0, −2)
77–78	Thalamus	Tha	Subcortical	(8, −17, 6) – (−14, −18, 6)
79–80	Heschl's gyrus	Heschl	Primary	(39, −16, 9) – (−47, −18, 8)
81–82	Superior temporal gyrus	STG	Association	(53, −21, 6) – (−56, −21, 5)
83–84	Temporal pole (superior)	TPOsup	Paralimbic	(43, 15, −19) – (−44, 15, −24)
85–86	Middle temporal gyrus	MTG	Association	(53, −37, −2) – (−59, −34, −5)
87–88	Temporal pole (middle)	TPOmid	Paralimbic	(40, 14, −34) – (−40, 13, −37)
89–90	Inferior temporal gyrus	ITG	Association	(49, −32, −23) – (−53, −29, −26)

The second expression was simplified using a notation similar to MATLAB vector syntax, in which *x*(*n* : *m*) represents the portion of *x* ranging from the *n*-th to the *m*-th entries (i.e., the portion of the time series *x* ranging from the *n*-th to the *m*-th measurement). Thus, *C*_*ij*_(*t*) is the linear correlation between *x*_*i*_ and *x*_*j*_ during a window of length *k* starting from *t*. As mentioned above, *C*_*ij*_(*t*) was computed with *k* = 60, which corresponds to ≈2 min.

### 2.5. EEG power, motion, cardiac, and respiratory time courses

**Figure 1:**
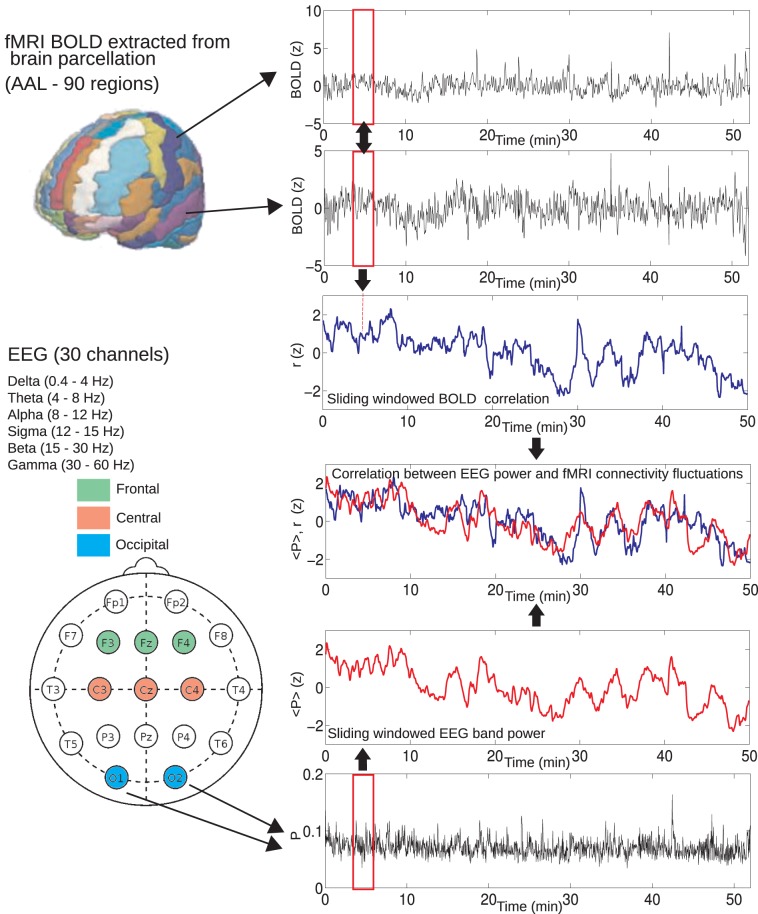
**Method used to compute BOLD connectivity fluctuations and correlations with EEG power fluctuations.** For each pair of regions, average BOLD signals were extracted and correlated using a sliding window of 60 volumes (≈2 min). This resulted in a connectivity estimate over time. A similar sliding window approach was used to obtain the average EEG power from different frequency bands (delta, theta, alpha, sigma, beta, and gamma), averaged from different locations (frontal, central, and occipital). As a final step, these EEG power fluctuations were correlated with BOLD connectivity for each pair of regions and correlations were tested for statistical significance (Student's *t*-test, FDR controlled for multiple comparisons).

Next, time courses for the variables to be correlated with BOLD connectivity were obtained. For this purpose, a sliding window average was applied, with the same window length (≈2 min) used to construct the time-dependent functional connectivity matrix (*C*_*ij*_(*t*)). Given a time series *y*, the computation is as follows:

(2) $$Y(t)=\frac{1}{k}\sum_{n=t}^{t+k}y(n)=\langle y(t:t+k)\rangle$$

which gives the desired result when *k* = 60. These sliding window-averaged time courses were obtained for delta (0.4–4 Hz), theta (4–8 Hz), alpha (8–12 Hz), sigma (12–15 Hz), beta (15–30 Hz), and gamma (30–60 Hz) power, averaged over frontal (channels F1, Fz, and F2), central (channels C1, Cz, and C2), and occipital (channels O1, Oz, and O2) EEG and also for the cardiac and respiratory time series estimated with the RETROICOR method. The sliding window-averaged time course for the relative displacement with respect to an arbitrary volume was also obtained, computed as $D=\sqrt{D_{x}^{2}+D_{y}^{2}+D_{z}^{2}}$, where *D*_x_, *D*_y_, and *D*_z_ are the estimated displacements (after spatial realignment with SPM8) in the *x*, *y*, and *z* axis, respectively. For an overview of the data analysis, see Figure 1.

### 2.6. Correlation between fMRI connectivity fluctuations and electrophysiological time series

Next, the time-dependent BOLD functional connectivity between each pair of regions [as represented in *C*_*ij*_(*t*)] was correlated with the sliding window-averaged time series of electrophysiological origin. Even though cardiac, respiratory and motion time series were regressed out of the signal at the pre-processing stage, they were still kept as partial regressors in the analysis. Therefore, we obtained the partial correlation between *C*_*ij*_(*t*) for 1 ≤ *i* ≤ 90, 1 ≤ *j* ≤ *i* − 1 and the different frequency bands from frontal, central and occipital channels:

(3) $$R_{C}(X,\;Y)=\underset{Z}{\min}R(X,\;Y|Z)$$

Here, *Z* runs through all the other variables involved in the partial correlation. *R*(*X*, *Y*|*Z*) is defined as follows,

(4) $$R(X,\;Y|Z)=\frac{R(X,\;Y)-R(Y,\;Z)}{\sqrt{1-R{\left(X,\;Z\right)}^{2}}\sqrt{1-R{\left(Y,\;Z\right)}^{2}}}$$

in which *R*(*X*, *Y*) is the linear correlation between both variables, as in Equation (1).

### 2.7. Time-dependent graph metrics

Then we analyzed the presence of correlations between the time development of common graph metrics associated with the global fMRI functional connectivity networks and time courses of EEG power fluctuations in different frequency bands. Graph metrics summarize topological information (i.e., not explicitly related to brain anatomy or geometry) about brain connectivity. For this purpose, a graph representation of functional connectivity is needed, in which each *node* represents a brain anatomical region and a *link* between two nodes represents significant functional connectivity between the BOLD signals from the nodes. The study of these metrics allows to map changes in global connectivity architecture [important to understand the information processing capacities of the system (Bullmore and Sporns, 2012)] related to a re-organization of the strongest connections. To obtain this representation, the correlation matrix *C*_*ij*_(*t*) was thresholded to obtain the time-dependent adjacency matrix (Bassett et al., 2010) *A*_*ij*_(*t*) as follows:

(5) $$A_{ij}(t)=\{\begin{array}{cc} 0 & \text{if }C_{ij}(t)<\rho \\ 1 & \text{if }C_{ij}(t)\geq \rho \end{array}$$

In the adjacency matrix *A*_*ij*_(*t*) a 1 represents a link between nodes *i* and *j* at time *t*. The arbitrary parameter ρ was chosen so that in all cases the resulting networks had a link density of 0.10, i.e., 10% of the total number of possible links in the networks were actually present. For each time step, a number of graph metrics using the MATLAB Brain Connectivity Toolbox (Rubinov and Sporns, 2010) was computed. Heuristic definitions are provided below [illustrations exemplifying the different metrics can be found in Figure 10A, for a detailed review see Sporns et al. (2004), Bullmore and Sporns (2009), and Rubinov and Sporns (2010)]:

- *Clustering coefficient (γ)*. The clustering coefficient of a given node is the probability that two nodes which are connected to it, are connected to one another. The clustering coefficient is then computed as the average of the clustering coefficient of all individual nodes.
- *Average path length (λ)*. The distance between node *i* and node *j* is the minimum number of links which have to be crossed when going from *i* to *j*. The average path length is the average of the distance between all possible pairs of nodes in the network.
- *Betweeness (β)*. A path between node *i* and node *j* is defined as the sequence of linked nodes which have to be visited to go from *i* to *j*. A minimum path between node *i* and node *j* is a path with a number of links equal to the distance between *i* and *j*. The betweeness of a given node in the network is defined as the number of minimum paths of which that node is a member. The betweeness of the network is computed as the average betweeness of all individual nodes.
- *Small-worldness (σ)*. To compute the small-worldness coefficient, networks are first randomized, scrambling their links at random with the constraint of a preserved connectivity distribution. Then, the clustering coefficient (γ_Rand_) and the average path length (λ_Rand_) of the randomized networks are computed. The small-worldness coefficient is then obtained as $\sigma =\frac{\gamma^{*}}{\lambda^{*}}$, where $\gamma^{*}=\frac{\gamma }{\gamma_{\text{Rand}}}$ and $\lambda^{*}=\frac{\lambda }{\lambda_{\text{Rand}}}$. A value of σ > 1 is regarded as indicator of small-world structure in the network (Humphries et al., 2006).

After obtaining the time courses of γ(*t*), λ(*t*), β(*t*), and σ(*t*), the presence of correlations with time courses of EEG power in the delta, theta, alpha, sigma, beta, and gamma bands was studied (taking cardiac, respiratory, and motion time series into account as partial regressors).

### 2.8. Statistical testing

To test for statistical significance, all correlation values were first transformed to z-scores using the Fisher transform, given by *z* = artanh(*r*). An ANOVA test was used to study the effect of EEG frequency band on the correlation coefficients with BOLD connectivity time courses. Then, Student's *t*-tests were performed with the null hypothesis of zero correlation. To correct for the multiple comparisons performed the False Discovery Rate (FDR) method was used, with *q* = 0.05. For the correlation between graph metrics and EEG power time courses Bonferroni correction was applied with *n* = 6 (frequency bands) × 3 (number of channel groups) × 4 (number of graph metrics) = 72.

## 3. Results

### 3.1. Dynamic spontaneous bold connectivity fluctuations

We started by assessing the presence of temporal fluctuations in BOLD connectivity, a necessary first step to perform the correlation analysis with EEG power fluctuations.

**Figure 2:**
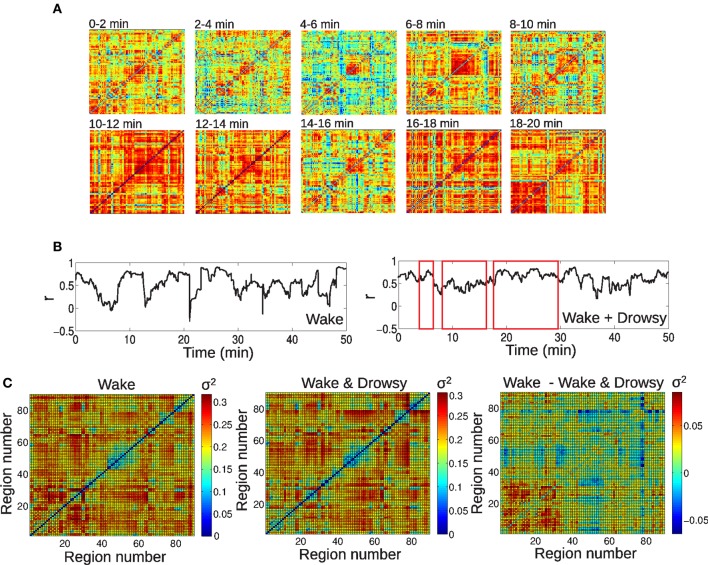
**Large-scale spontaneous BOLD functional connectivity fluctuations. (A)** BOLD correlation matrices for a single subject, in intervals of 2 min. **(B)** Time series of inter-hemispheric thalamic connectivity for an awake subject and a subject undergoing vigilance transitions between wakefulness and light sleep (periods of light sleep are marked by red rectangles). **(C)** Standard deviation of BOLD connectivity time series for each pair of regions, for both groups (wakefulness and wakefulness & light sleep) and their difference. For the AAL regions associated with region numbers, see Table 1.

Functional connectivity between brain regions fluctuated widely over time, consistently with previous reports (Chang and Glover, 2010; Handwerker et al., 2012; Hutchison et al., 2012). An example of this dynamic functional connectivity is shown in Figure 2A, in which the complete functional connectivity matrix of a single subject is presented in intervals of 2 min. It is clear, by simple visual inspection, that the connectivity matrix fluctuates over time, with periods of overall decreased connectivity alternating with periods of globally increased connectivity [termed *hypersynchrony* in a previous study (Hutchison et al., 2012)]. As another example, in Figure 2B the time courses of connectivity between left and right thalamus are shown for the same subject and also for a subject undergoing vigilance transitions between wakefulness and light (N1) sleep. Correlation between the homeotopic BOLD signals clearly changes over time, alternating between a correlation close to the highest possible value (*r* = 1) and a complete discoordination (*r* = 0), in spite of strong average inter-thalamic connectivity (*r* > 0.5). The extent of functional connectivity variation was quantified by taking the standard deviation (SD) of the connectivity time course between all pairs of regions. The average SD for both groups are shown in Figure 2C, together with their difference. “Blocks” of lower SD can be observed grouped around the diagonal of the matrix, indicating groups of (anatomically) neighboring regions with lower functional connectivity variability over time (for example, occipital regions, ranging from regions #43–44 to #55–56), while displaying a higher variability in their connections with the rest of the brain (off-diagonal entries). While there were no significant differences surviving multiple comparison correction, there was a trend of higher SD in frontal connectivity for the group of awake subjects and of higher SD in cortico-thalamic connectivity for the group of subjects exhibiting vigilance transitions between wakefulness and light sleep.

### 3.2. Correlations with spontaneous EEG power fluctuations

**Figure 3:**
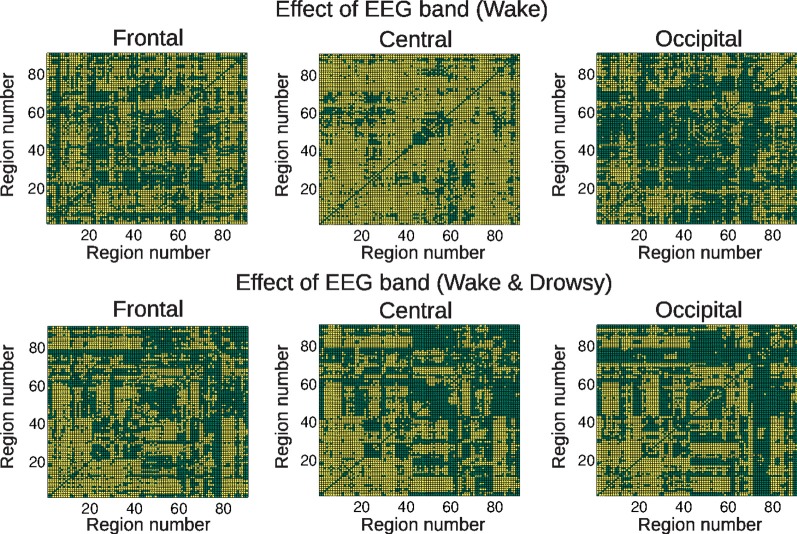
**Connections showing a significant (colored in yellow) effect of EEG frequency band in the correlation coefficient between BOLD functional connectivity and spontaneous EEG power fluctuations, for the three scalp regions defined in Figure 1 (frontal, central, and occipital) and both groups (wakefulness and wakefulness & light sleep).** For the AAL regions associated with region numbers, see Table 1.

Next, we studied the correlations between EEG power and BOLD connectivity time courses, in order to identify EEG frequency bands involved in connectivity changes over time. We first studied the effect of the different EEG frequency bands on the correlation coefficient with dynamic BOLD connectivity. Results for both groups are presented in Figure 3, in which a frequency-dependent effect can be readily appreciated. In what follows, we explore these correlations specific to each frequency band, topographic location, and group of subjects.

**Figure 4:**
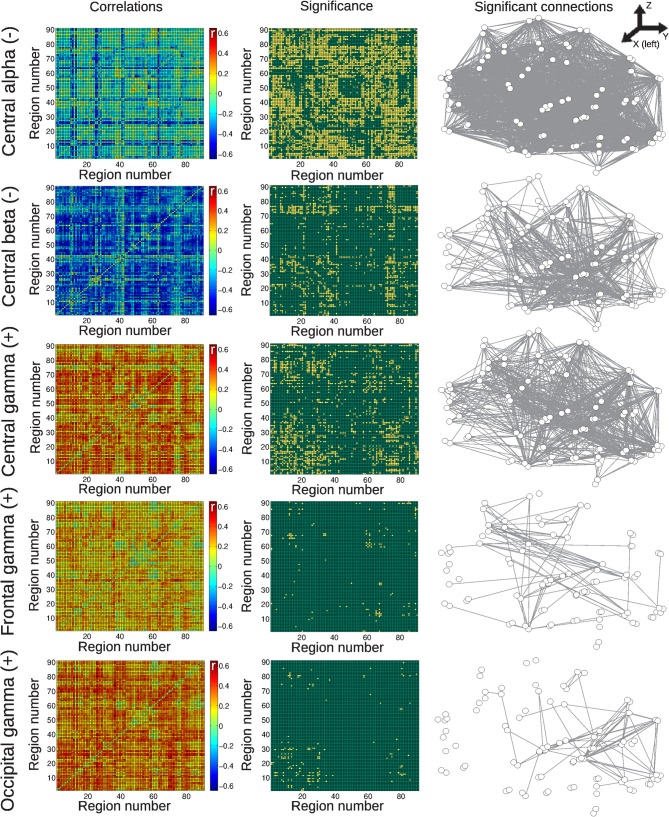
**Matrices of average correlation (left), significant correlations (center, significant correlations in yellow), and networks in anatomical space (left: posterior side, right: anterior side) with links representing significant correlations (right).** Correlations are between temporal changes in BOLD functional connectivity and changes in EEG power, for all frequency bands and averaged from different anatomical locations (see “Materials and Methods”). Results are for the group of awake subjects. For the AAL regions associated with region numbers, see Table 1.

Results for the group of awake subjects are presented in Figure 4. Widespread negative correlations between BOLD connectivity fluctuations and central alpha and beta power were detected. Positive correlations were found with central, frontal, and occipital gamma power fluctuations, with correlations with central channels being more widespread than the others.

**Figure 5:**
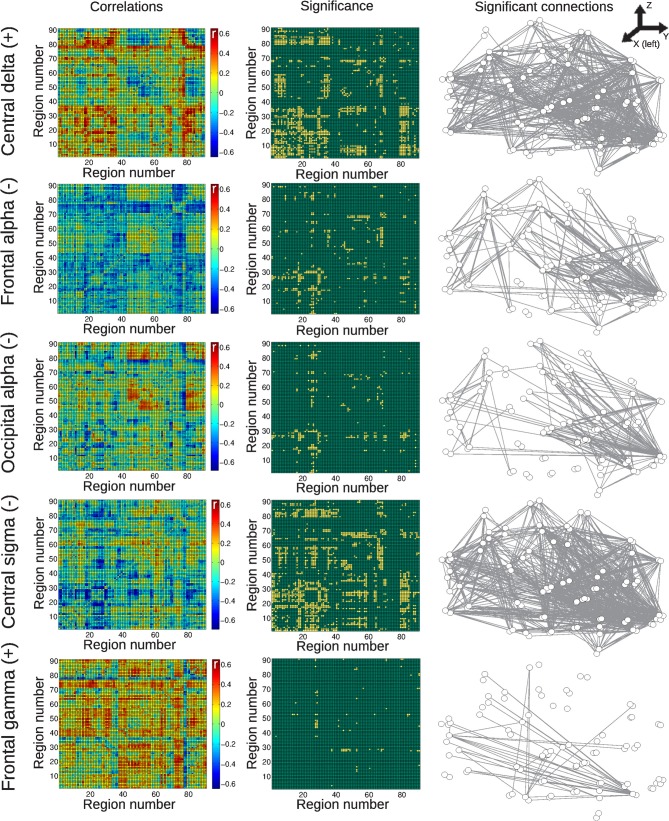
**Matrices of average correlation (left), significant correlations (center, significant correlations in yellow), and networks in anatomical space (left: posterior side, right: anterior side) with links representing significant correlations (right).** Correlations are between temporal changes in BOLD functional connectivity and changes in EEG power, for all frequency bands and averaged from different anatomical locations (see “Materials and Methods”). Results are for the group of subjects undergoing vigilance transitions between wakefulness and light sleep. For the AAL regions associated with region numbers, see Table 1.

Correlations for the group of subjects undergoing vigilance changes are presented in Figure 5. Positive correlations were found with central delta power fluctuations, with a spatial emphasis in frontal regions and between frontal and temporal regions. Negative correlations with frontal and occipital alpha also affected predominantly frontal BOLD connectivity, whereas the central sigma band showed more distributed negative correlations. Finally, very few positive correlations with the frontal gamma time course were found.

We also directly tested whether functional connectivity changes correlated with cardiac, respiratory, and motion time series. Negative results were found for cardiac and motion time series, and only spurious positive correlations for the group of awake subjects (four pairs of regions) and for the subjects with fluctuating vigilance (two pairs of regions).

### 3.3. Nodes with connectivities most influenced by EEG power fluctuations

To extract information about the network of connections correlated with EEG power in specific frequency bands, we computed for each node the number of connections with other nodes which were affected by spontaneous power fluctuations. This is equivalent to the degree (D) of each region or node, if one defines a network whose links are the EEG power-modulated connections.

**Figure 6:**
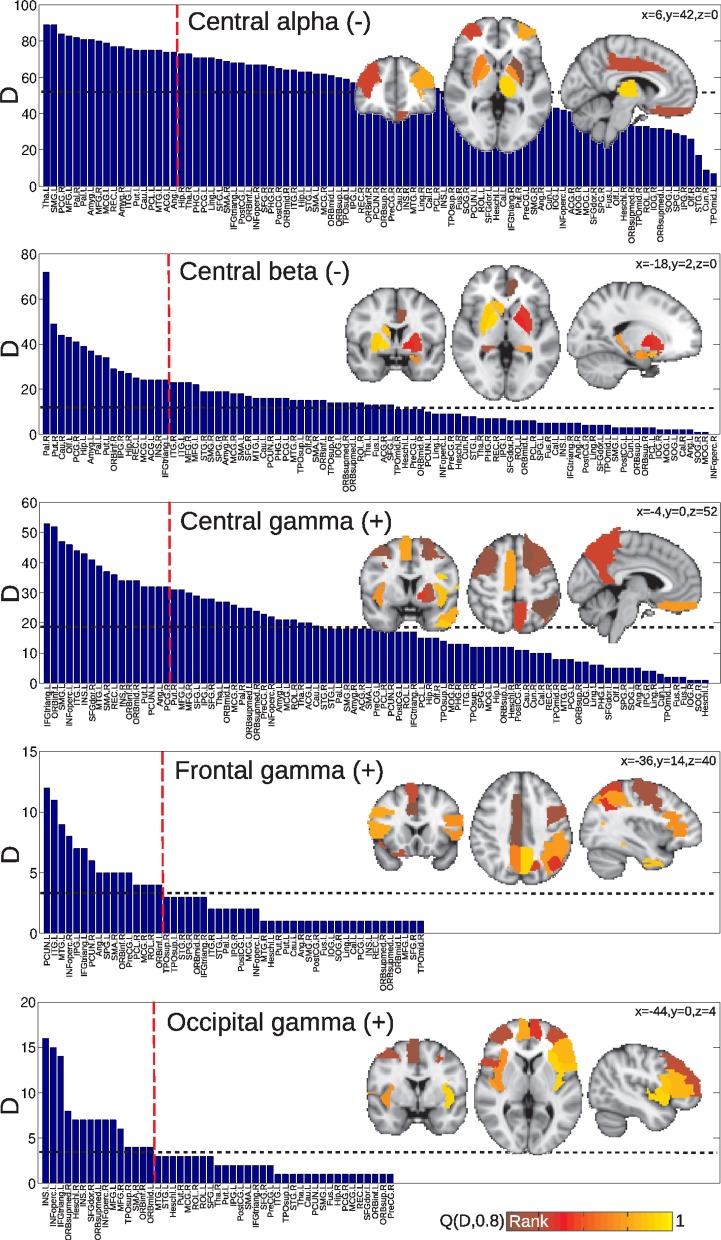
**Anatomical regions (or nodes) ranked according to the number of connections attached to them which correlate with EEG power fluctuations in different frequencies.** In the inset, the regions corresponding to the top quintile of the distribution—denoted as *Q*(*D*, 0.8)—are displayed overlaid on a standard MNI T1 template. The horizontal dashed line indicates the mean of the distribution. Results are for the group of awake subjects.

**Figure 7:**
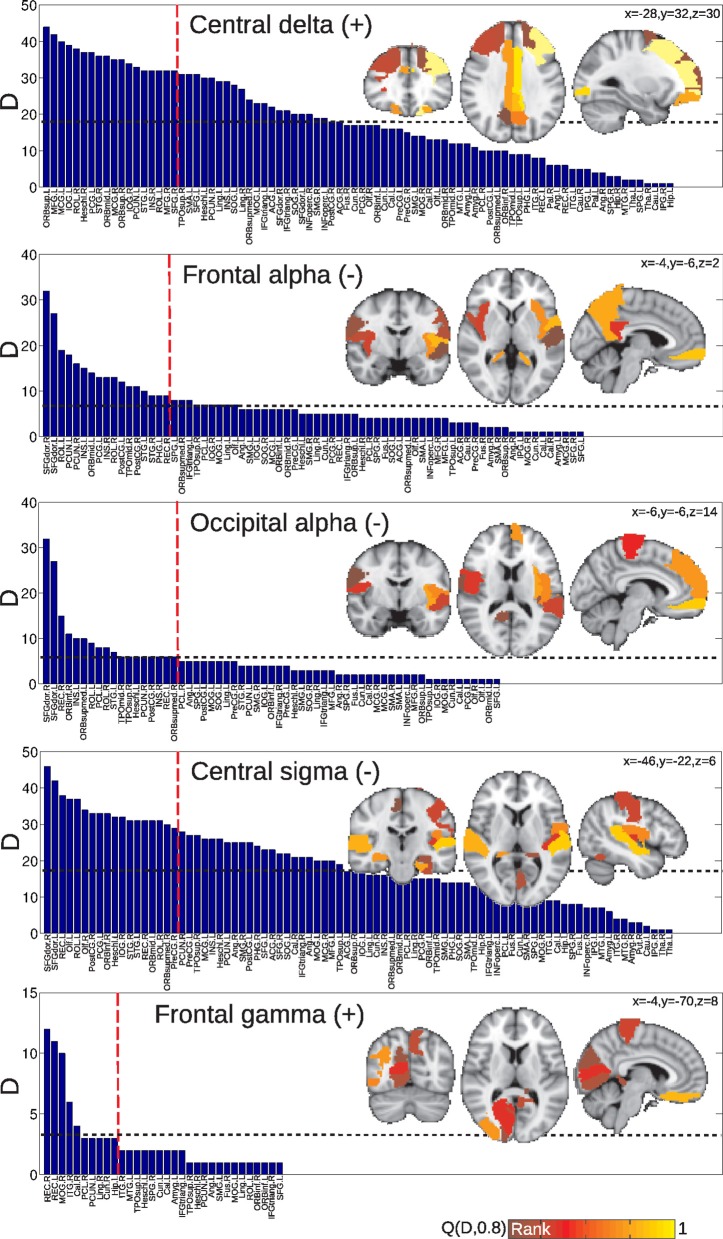
**Anatomical regions (or nodes) ranked according to the number of connections attached to them which correlate with EEG power fluctuations in different frequencies.** In the inset, the regions corresponding to the top quintile of the distribution—denoted as *Q*(*D*, 0.8)—are displayed overlaid on a standard MNI T1 template. The horizontal dashed line indicates the mean of the distribution. Results are for the group of subjects undergoing vigilance transitions between wakefulness and light sleep.

After having obtained the degree for each region, areas were ranked in decreasing order. Results are shown in Figure 6 for the group of awake subjects and Figure 7 for the group of subjects undergoing vigilance transitions. In the insets, regions corresponding to the 4th quintile of the distributions are displayed overlaid on a standard MNI T1 template (lighter colors represent a higher number of correlations). For the group of awake subjects, the region with the highest number of negative correlations with central alpha was the thalamus. Other top-ranked regions were sub-cortical and bilateral frontal. For correlations with beta, highest ranked regions were also subcortical (pallidum, putamen and caudate nucleus). Top-ranked regions with positive functional connectivity correlations in the central gamma band were mostly frontal. The same was observed for correlations with occipital gamma (with the inclusion of the bilateral insular cortices as the regions with the highest degree). In correlations with frontal gamma, on the other hand, frontal regions were second to precuneus, temporal and parietal areas (Figure 6).

For the group of subjects undergoing vigilance transitions, superior and middle frontal gyri and cingulate regions exhibited the largest number of positive correlations with delta power. The same was observed for negative correlations with frontal and occipital alpha (including also insular and precuneal cortices) and central sigma (in all three cases, the top-ranked region was the dorsal part of the superior frontal gyrus). The top-ranked region for positive correlations with frontal gamma was the gyrus rectus followed by occipital regions.

### 3.4. EEG power fluctuations and connectivity between different sets of brain regions

We quantified the number of connections modulated by EEG power between different sets of brain regions, in order to reveal frequency-specific changes in communication between brain systems.

**Figure 8:**
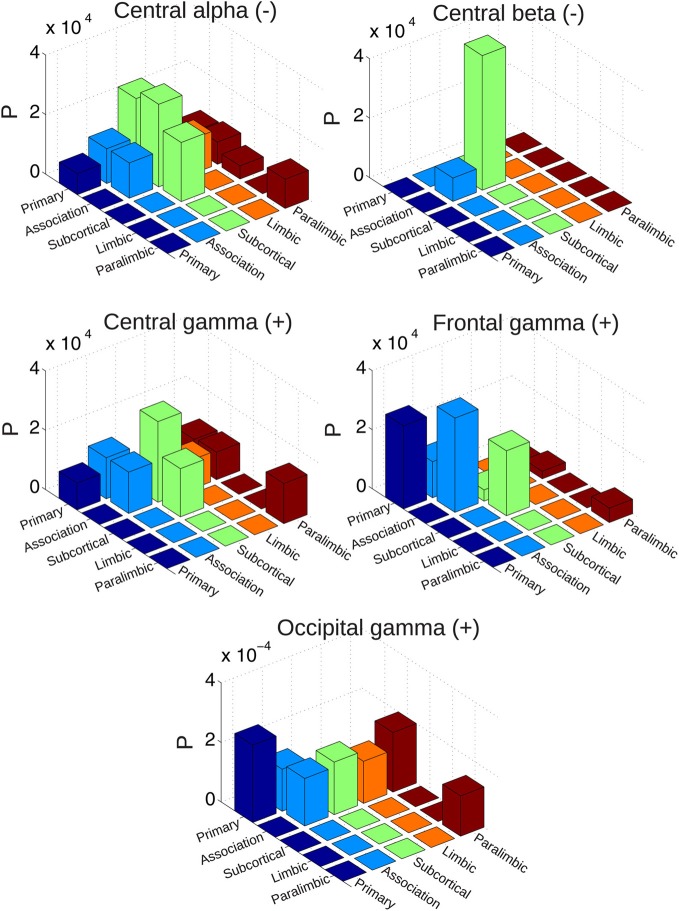
**Probability of finding connections between different systems (sensory, association, subcortical, limbic, and paralimbic) which correlate either positively or negatively with spontaneous EEG power fluctuations (normalized by the total number of possible connections between each pair of systems).** Results are for the group of awake subjects.

**Figure 9:**
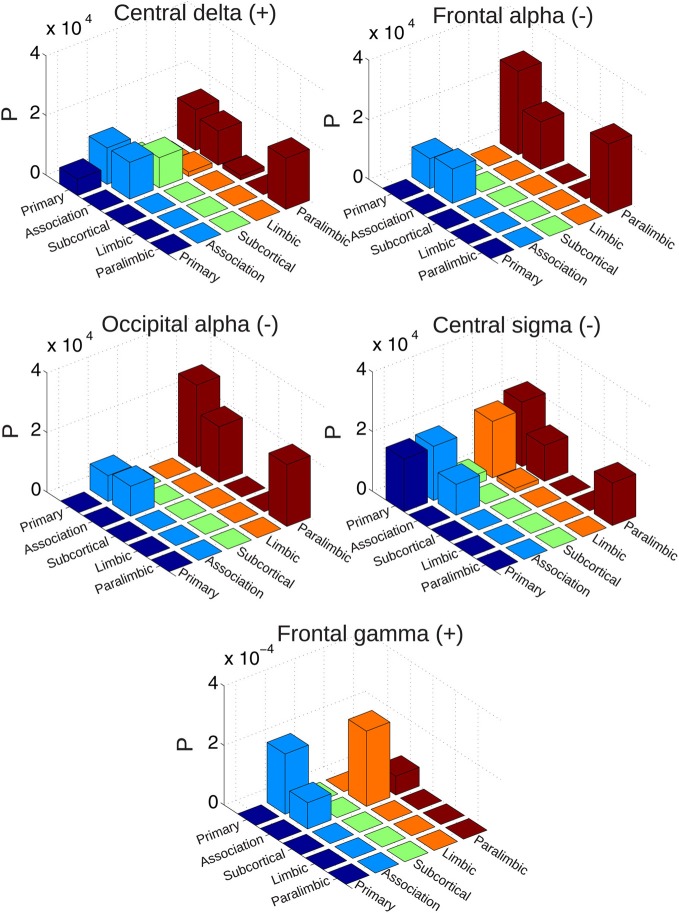
**Probability of finding connections between different systems (sensory, association, subcortical, limbic, and paralimbic) which correlate either positively or negatively with spontaneous EEG power fluctuations (normalized by the total number of possible connections between each pair of systems).** Results are for the group of subjects undergoing vigilance transitions between wakefulness and light sleep.

For that purpose, a previously introduced classification of each region into five categories was followed, comprising primary sensory, association, subcortical, limbic, and paralimbic areas (Achard et al., 2006) (the system membership of AAL regions can be found in Table 1). The *network traffic* between each pair of systems was computed as the normalized number of connections which covary negatively or positively with spontaneous EEG power fluctuations. Results (normalized by the total number of possible connections between each pair of systems) are shown in Figure 8 for the group of awake subjects and in Figure 9 for the group of subjects undergoing wakefulness-light sleep transitions. In the first group, EEG alpha power mediated decreased BOLD connectivity mostly between subcortical areas, association and primary cortices. The effect of central beta was marked by changes affecting almost solely connections between subcortical and association areas. Positive correlations with central gamma were more widespread but again peaking at the interaction between subcortical and association systems. A similar effect was observed for correlations with occipital gamma (affecting connections between association areas and all other systems). For frontal gamma power, however, we observed increased BOLD connectivity inside (and not between) primary, subcortical and association areas. For the second group (wakefulness and light sleep) the most salient feature was an involvement of paralimbic connectivity between primary and association cortices. Also, connectivity inside the association system and with the primary cortex was affected for all frequency bands considered.

### 3.5. Correlation between EEG power and spontaneous graph metric fluctuations

The observed correlations between EEG power fluctuations and BOLD connectivity suggest that graph theoretical measures of network organization should also covary with EEG power changes. We studied this possibility for the common graph metrics, clustering coefficient, average path length, betweeness, and small-worldness.

**Figure 10:**
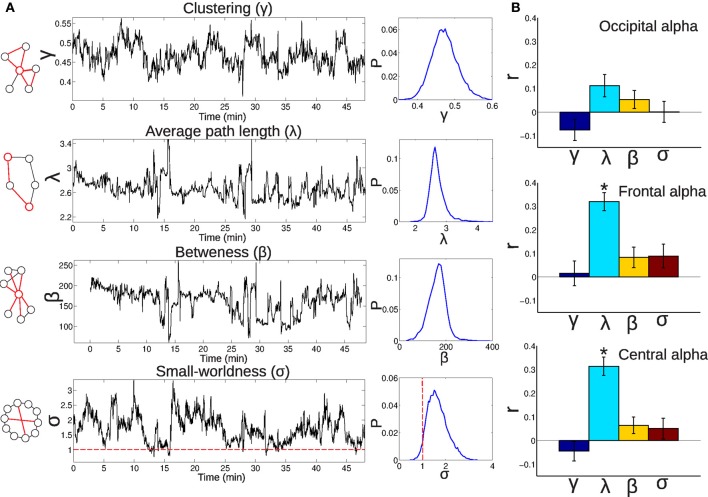
**(A)** Left: Illustrations exemplifying the meaning of common graph metrics (clustering coefficient, average path length, betweeness, and small-worldness; for a detailed explanation, see the “Materials and Methods” section). Center: Examples showing the temporal evolution of the graph metrics for a single subject. Right: Probability (*P*) distributions (for all subjects) of the graph metric values. **(B)** Correlations between fluctuations in the graph metrics and EEG alpha power, averaged from occipital, frontal, and central electrodes (^*^significant at *p*<0.05, Bonferroni corrected, *n* = 72).

In Figure 10A (left) the definitions provided in the “Materials and Methods” section are illustrated and an example of how the different graph metrics change during the duration of a single subject measurement (Figure 10A, center) is provided, together with histograms of graph metric values for all subjects (Figure 10A, right). Note that while small-worldness (σ) is usually greater than 1 [the limit value at which networks are usually considered to be small-world (Humphries et al., 2006; van den Heuvel et al., 2008)], at certain times this value goes below this threshold, highlighting the fact that the claim of small-worldness of brain functional networks originates from average connectivity, but that in fact small-worldness indeed is not given at all times. It must be noted that the use of Pearson correlation to compute functional connectivity networks will result in an overestimation of the number of triangles (i.e., the clustering coefficient) and thus also of the small-worldness (for a discussion see Smith et al., 2011). An overestimation of this index, however, is likely to leave relative differences unaffected, preserving the dynamics. In Figure 10B, the temporal correlations between fluctuations in graph metrics and occipital, frontal and central EEG alpha power are presented (the other frequency bands did not exhibit significant correlations). These correlations were positive between average path length and frontal-central alpha. An increased average path length signals a more fragmented network, consistent with the widespread BOLD discoordination observed at times of high alpha power (Figure 4).

## 4. Discussion

In this work we have studied how changes over time in BOLD functional connectivity are linked to increased local synchronization of scalp EEG rhythms, as indexed by spectral power. Our results reveal that increased EEG power in the gamma band facilitated long-range communication between brain regions (especially for those located in frontal areas), whereas frequencies in the alpha and beta range were related to diminished functional connectivity. When studied using a graph theoretical approach, these results were manifest in a positive correlation between power in the alpha band and average path length, compatible with the interpretation of less efficient connectivity between regions.

Our results are concordant with a recent study of primary visual cortex connectivity modulation by spontaneous power fluctuations in posterior alpha (Scheeringa et al., 2012), and represent an extension from regional and system-specific correlations to the global relationship between EEG frequency power changes and BOLD connectivity. Another recent study addressed the opposite question: is BOLD amplitude correlated with changes in alpha EEG phase synchronization? (Sadaghiani et al., 2012). Positive correlations between global synchronization and fMRI signal amplitude were found in a fronto-parietal network of regions, favoring an interpretation of alpha phase locking as related to integration of information in this cortical network. However, our direct evaluation of alpha power influence on BOLD connectivity suggests that periods of high alpha are characterized by a widespread disruption of BOLD coherence, which could be related to inhibitory process facilitating information flow in the unaffected nodes (Klimesch et al., 2007; Jensen and Mazaheri, 2010) (see discussion below).

In the following paragraphs we discuss in detail our results in the light of previous work and theories about brain function, and we develop the most important implications of our results for future studies of brain functional connectivity.

### 4.1. Bold functional connectivity fluctuates at a time scale of minutes

Our sliding window analysis revealed changes in functional connectivity at the scale of minutes. Such non-stationary connectivity can in principle be attributed to many factors or combinations of factors: intrinsic coupling and de-coupling of neural activity during rest, vigilance changes, spontaneous cognition, movement artifacts, and changes in cardiac or respiratory rates. A previous study demonstrated temporal changes in connectivity (termed dynamic functional connectivity) in anesthetized macaques while ruling out motion and spontaneous conscious cognition as the origin of the observed BOLD connectivity changes (Hutchison et al., 2012). Our study does not eliminate spontaneous cognition, rather, our EEG-fMRI analysis allows to map connectivity changes correlated with the power of scalp oscillations involved in different brain states and cognitive processes. Cardiac and motion time series did not correlate with changes in functional connectivity, and respiratory time series only correlated with a minuscule fraction of the possible connections. A large number of correlations with different EEG frequencies (discussed in detail below)—present even when motion, cardiac, and respiratory time series were taken into account as partial regressors—highlight the neural origin of the changes in BOLD connectivity: the presence of specific neural oscillations is directly related to increased or decreased temporal synchronization of BOLD signals from distinct cortical and subcortical areas.

Furthermore, the SD of sliding windowed BOLD connectivity, which quantifies the degree of deviation from a stationary connectivity, displayed structured spatial variation: connections between neighboring regions had a smaller SD, while connections between regions from heterogeneous systems showed larger deviations from constant connectivity.

Finally, our analysis included two groups of subjects: one showing steady levels of vigilance (wakefulness) and the other with transitions between wakefulness and light sleep. The onset of sleep is characterized by changes in scalp EEG oscillations [a shift from fast toward slower frequencies (AASM, 2007)]. Our analysis revealed that BOLD connectivity correlates with these spectral changes in the group of subjects falling asleep. Distributed positive temporal correlations between BOLD connectivity and EEG power in the delta band appeared, while positive correlations with the gamma band mostly disappeared. Our results also suggest an electrophysiological correlate of changes in BOLD connectivity as previously reported at sleep onset (Spoormaker et al., 2010; Tagliazucchi et al., 2012).

### 4.2. Negative correlations with EEG power fluctuations

We have shown that spontaneous increases in EEG alpha power (averaged over central channels) correlates with decreased BOLD connectivity between a large number of cortical and subcortical regions. This would be consistent with the proposition of alpha as an “idling rhythm” which predominates during relaxed, eyes closed rest (Pfurtscheller et al., 1996) and with that of alpha as performing functional inhibition of regions not relevant to task performance (Klimesch et al., 2007; Jensen and Mazaheri, 2010; Scheeringa et al., 2012). The BOLD connectivity decreases observed at times of large alpha amplitude could indicate an active suppression of sensory input and its subsequent cortical processing (Worden et al., 2000). While alpha is usually more prominent in occipital regions, the “blocking” of oscillations in the same frequency range has been related to the onset and planning of activity in sensory-motor and supplementary motor cortices (Pfurtscheller et al., 1997) [these oscillations in the alpha range are termed “mu” or “rolandic alpha rhythm” (Pfurtscheller and Andrew, 1999)]. Furthermore, previous EEG-fMRI studies demonstrated an inverse relationship between BOLD activation in a large, distributed network of cortical areas (Laufs et al., 2003a; Moosmann et al., 2003; Goncalves et al., 2006) [overlapping with the default mode network (Laufs et al., 2003b)] and EEG alpha power. Overall, these results suggest that alpha suppression is a landmark of cortical activation, a view which is expanded by the present work demonstrating a direct link with increased BOLD connectivity. Vice versa, during epochs of increased alpha oscillations, functional connectivity is—efficiently—temporally weakened.

The negative correlations obtained between BOLD connectivity and beta power are at first sight surprising, given the traditional view of these faster rhythms as a signal of increased mental activity, starting from their first observations by (Berger, 1938). However, desynchronization of rolandic (peri-central) beta rhythms increases cortical excitability, favoring a motor response (Deletis et al., 1992). Beta rhythms over central regions appear in synchronized fashion after (but not during) the execution of a voluntary motor command (Pfurtscheller, 1981; Pfurtscheller et al., 2003). Because of this inverse relationship between rolandic beta rhythms and cortical excitability they are, like alpha, usually considered as idling rhythms, which indicate a resting state of the sensory-motor cortex and related brain areas (Pfurtscheller and Lopez da Silva, 1999). Furthermore, an inverse relationship between peri-central beta rhythms and fMRI BOLD activation has been reported (Ritter et al., 2008). Considered together, these results suggest that both rolandic beta and alpha rhythms are related to an idling state of the cortex, which we demonstrate here related to a decreased BOLD functional connectivity. While in our study subjects did not perform any explicit motor task, we hypothesize that during rest brain activity spontaneously recapitulates activity patterns related to the execution of diverse tasks, as well as stimuli perception—a hypothesis which has received strong experimental support from different neuroimaging modalities (Ringach, 2009; Smith et al., 2009; Sadaghiani et al., 2010).

We also note that the connectivity of subcortical regions was most strongly affected by central and alpha rhythms, while primary sensory and motor cortices remained relatively unaffected. This suggests that the presence of idling rhythms in scalp EEG is related to a loss of functional connectivity between cortical areas and specific subcortical structures (e.g., thalamus for alpha rhythm and basal ganglia for beta rhythm). As an example, since spontaneous thalamic BOLD amplitude has been shown to correlate positively with EEG alpha power, and activity from a large network of cortical areas shows an inverse correlation (Laufs et al., 2003b), we can expect compromised cortico-thalamic BOLD connectivity modulations to be linked to spontaneous changes in alpha power. This is also supported by our analysis of alpha power and connectivity between different brain systems. As shown in Figure 8, the connectivity between subcortical regions and primary and association cortices [which include the regions reported in Laufs et al. (2003b)] is most compromised during periods of high alpha power. Subjects undergoing vigilance changes consistently displayed negatively correlated connectivity with alpha and sigma bands in frontal regions (bilateral superior frontal gyrus), which could be related to vigilance-related variability in correlation patterns with alpha power (Laufs et al., 2006).

### 4.3. Positive correlations with EEG power fluctuations

In contrast to the slower frequencies, the faster gamma rhythm is almost universally related to cognitive performance. Since the first pioneering studies showing increased gamma amplitude and synchronization during visual stimulation (Singer et al., 1997), a wealth of experimental results has demonstrated the important role of activity in the gamma band during the execution of different cognitive tasks (for reviews, see Lee et al., 2003; Herrmann et al., 2004; Fries, 2005; Fries et al., 2007). The ubiquity of gamma rhythms and their apparently heterogeneous nature have led to the hypothesis that activity in the gamma band represents a fundamental process subserving an elementary operation of cortical computation (Fries, 2009). Our results closely relate increased EEG gamma power (averaged from different topographical locations) to the coordination of BOLD signals between a large number of cortical and subcortical pairs of regions. If one accepts that spontaneous fluctuations in BOLD and EEG signals resemble elicited activity patterns, this result is consistent with the experiments and hypotheses mentioned above. Such similarity is supported by studies showing that spontaneous cognitive operations underlie resting state activity fluctuations (Andrews-Hanna et al., 2010; Shirer et al., 2012). This proposition, however, cannot be held as the only origin of the aforementioned fluctuations given the coordinated spatio-temporal activity observed in states of diminished conscious awareness, such as sleep (Boly et al., 2008; Horovitz et al., 2008; Larson-Prior et al., 2009; Brodbeck et al., 2012). An alternative interpretation of BOLD signal coherence and its electrophysiological correlates during the resting state is the setting up of a baseline state favoring a quick response to environmental demands (Buckner and Vincent, 2007). In such scenario spontaneous cognition does not cause the resemblance between elicited and spontaneous activity, but the need to be close at all times to a behaviorally meaningful state.

Connectivity of frontal, precuneal and temporal regions with the rest of the brain was most strongly influenced by the increase of gamma power. EEG-fMRI studies have shown that correlations between scalp EEG gamma and BOLD activity are located predominantly in frontal regions (Mantini et al., 2007). Our study extends this observation by demonstrating a direct relationship between increased gamma and BOLD connectivity of frontal regions with the rest of the brain. Increased gamma power was related to strengthened BOLD connectivity between association, primary, and subcortical regions (Figure 8), a fact consistent with responses in the gamma band observed during cross-modal processing (Kisley and Cornwell, 2006; Yuval-Greenberg and Deouell, 2007; Senkowski et al., 2008).

Also, we observed positive correlations between increased delta power and BOLD connectivity, but only for the subjects undergoing vigilance transitions to light sleep. The nature of scalp oscillations in the delta range is very different from those in the gamma band: their low temporal complexity reflects the alternation between neural firing (“up” state) and quiescence (“down” state). Such lack of temporal complexity, and therefore a diminished repertoire of possible neural states, was hypothesized to underlie loss of conscious awareness during sleep (Tononi et al., 1994; Tononi, 2004, 2008). Our results show that increased BOLD connectivity (suggestive of an excessive integration and a loss of functionally segregated brain modules) follows activity in the delta band for subjects transitioning to light sleep. Studies addressing the issue of temporal complexity (or temporal dependency) of BOLD signals across the human sleep cycle are needed to reveal in fMRI recordings the temporal properties of these slow neural oscillations.

### 4.4. Comparison between groups

The case for a neurophysiological origin of dynamical functional connectivity is supported by the correlations with EEG spectral power. To strengthen this observation, a manipulation can be performed in one of the variables involved in the correlation and the effect on the other can be measured. We considered the changes in EEG spectral power occurring in the transition to light sleep as such a manipulation. We indeed observed the expected changes in the correlation patterns. In contrast to the wakefulness group, the delta band was involved in strengthened BOLD connectivity, a fact expected given the general slowing of EEG frequencies which characterizes N1 sleep (see the AASM sleep scoring criteria in the “Materials and Methods” section). Increased connectivity associated with power in slow frequency bands could be related to the loss of consciousness during early sleep (see the last section of the “Discussion”). In the group of subjects undergoing transitions to light sleep, we also observed frontal and occipital alpha power to be correlated negatively with BOLD connectivity, as opposed to central alpha as in the group of awake subjects. This is expected given that the loss of alpha at sleep onset is observed predominantly in occipital channels (AASM, 2007), whereas alpha in central regions appears to be of a different nature and is associated with motor-related task demands (Pfurtscheller et al., 1997). Finally, the decreased and less widespread positive correlations with the gamma band are likely related to the loss of power in this frequency band during sleep. In particular, and as discussed in the previous section, increased BOLD connectivity of temporal, precuneal, and parietal areas during periods of high frontal gamma could relate to cross-modal binding. This effect was not observed in the group of subjects exhibiting light sleep, for whom primary sensory areas (visual) were mostly affected. A heterogeneous origin of gamma oscillations measured at these different behavioral states could underlie these differences.

### 4.5. Temporal scales and correlations between bold connectivity and EEG power

Given that changes in EEG power (for example, in beta or gamma bands) during or after task execution usually occur in the sub-second temporal range, it is remarkable that correlations with the connectivity of the slow, lagged, and relatively poorly sampled BOLD signal can be found. However, a period with a particularly high level of activity could elicit a change in BOLD connectivity when all the short-lived EEG power changes are considered together. This situation is analogous to that of the correlation between short periods of stable topographical configurations [EEG microstates (Koenig et al., 2002)] with specific BOLD RSN, which are likely driven by periods in which the presence of a given microstate overwhelmingly exceeds that of the others (Britz et al., 2010). It has been speculated and subsequently corroborated (Van De Ville et al., 2010) that, for this to happen, the distribution of the EEG events has to follow a scale-free distribution (or equivalently, have a 1/f spectrum): only then the temporal scale invariance allows the discovery of correlations using a much slower imaging method, such as fMRI. This scale invariance is a defining property of self-organized complex systems, like the brain (Tagliazucchi and Chialvo, 2011). 1/f spectra are ubiquitous in the power fluctuations of EEG, MEG, and electrocorticography (ECoG) recordings (Linkenkaer-Hansen et al., 2001; Miller et al., 2009; He et al., 2010; He, 2011), as well as in time series derived from cognitive and behavioral experiments (Gilden et al., 1995; Shelhamer and Joiner, 2003).

### 4.6. Graph metrics fluctuate over time

We have demonstrated that during the time evolution of whole-brain functional networks, associated graph metrics (clustering coefficient, average path length, betweeness, and small-worldness) also change over time. Given this result, a number of recent studies based on the methodology of graph theory have to be re-interpreted. The reported value of the different graph metrics cannot be taken as a constant property of brain networks, instead, it has to be considered as an asymptotic property (i.e., the value one obtains during a long measurement) emerging after temporal averaging. There are two interesting immediate consequences of this observation. First, resting state studies applying graph theoretical methods should be based on long recordings, since short acquisition times will decrease the confidence on the graph metric estimates (the risk of computing them in a period in which they largely deviate from the mean will be higher). Second, the temporal evolution of graph metrics should be taken into account. For example, when comparing two populations using graph theoretical methods, equal values of the associated graph metrics may be obtained, yet their dynamical behavior could be completely different. Further investigations are needed to study this and other possibilities of considering the evolution of functional networks over time.

### 4.7. Implications for theories of conscious brain function

Our results show that the onset of specific (fast) rhythms is accompanied by distributed binding of BOLD activity (i.e., increased functional connectivity), whereas other (slower) oscillations rather inhibit such binding, decreasing the overall cortico-cortical and cortico-subcortical connectivity. The fact that long-range functional connectivity in the human brain is unstable and fluctuates in a coordinated fashion with fast EEG rhythms likely reflects the dynamic nature of processes underlying the conscious state. For instance, an approach to consciousness focusing in its nature as a *process* (instead of a state or a capacity) emphasizes the presence of a *dynamical core*, a continuously changing set of neuronal groups strongly integrated together during hundreds of milliseconds and allowing differentiated responses, i.e., having a large neural complexity (Tononi and Edelman, 1998). Such complexity is hindered in cases of overly integrated or segregated dynamics, such as those present during sleep (Tononi et al., 1994; Tononi, 2004, 2008). In this framework, the higher large-scale BOLD functional connectivity associated with increased delta power could be related to the loss of consciousness which occurs during early sleep. Interestingly, an opposite scenario (increased functional segregation) was reported in a recent study (Boly et al., 2012). This seeming discrepancy could arise because all NREM sleep stages were considered together (including N2 and N3, indicating also deeper sleep).

While dynamical functional connectivity and its electrophysiological characteristics are suggestive of the processes postulated by the aforementioned theories, further experimental tests are required in order to corroborate them as a correlate of conscious awareness (for example, studying whether these interrelated, dynamical landmarks of brain activity are also prevalent during deeper sleep stages, anesthesia or coma).

### 4.8. Conclusion

Large efforts have been devoted to the identification of the electrophysiological correlates of the fMRI BOLD contrast. We have approached this problem from a new perspective: studying whether band-specific scalp oscillations are related to increased (or decreased) BOLD coherence instead of directly relating them to changes in BOLD amplitude. This approach has allowed us to observe, for the first time, a relationship between BOLD signal functional connectivity and increased local synchronization in the gamma band. Furthermore, slower “idling” rhythms were linked to large-scale disconnection patterns, also quantified by correlation with adequate graph metrics. The relationship found between EEG power fluctuations and dynamic BOLD functional connectivity leads us to conclude that this phenomenon is very likely to be of neuronal origin, and thus it deserves even further investigation.

### Conflict of interest statement

The authors declare that the research was conducted in the absence of any commercial or financial relationships that could be construed as a potential conflict of interest.

## Figures and Tables

**Figure A1:**
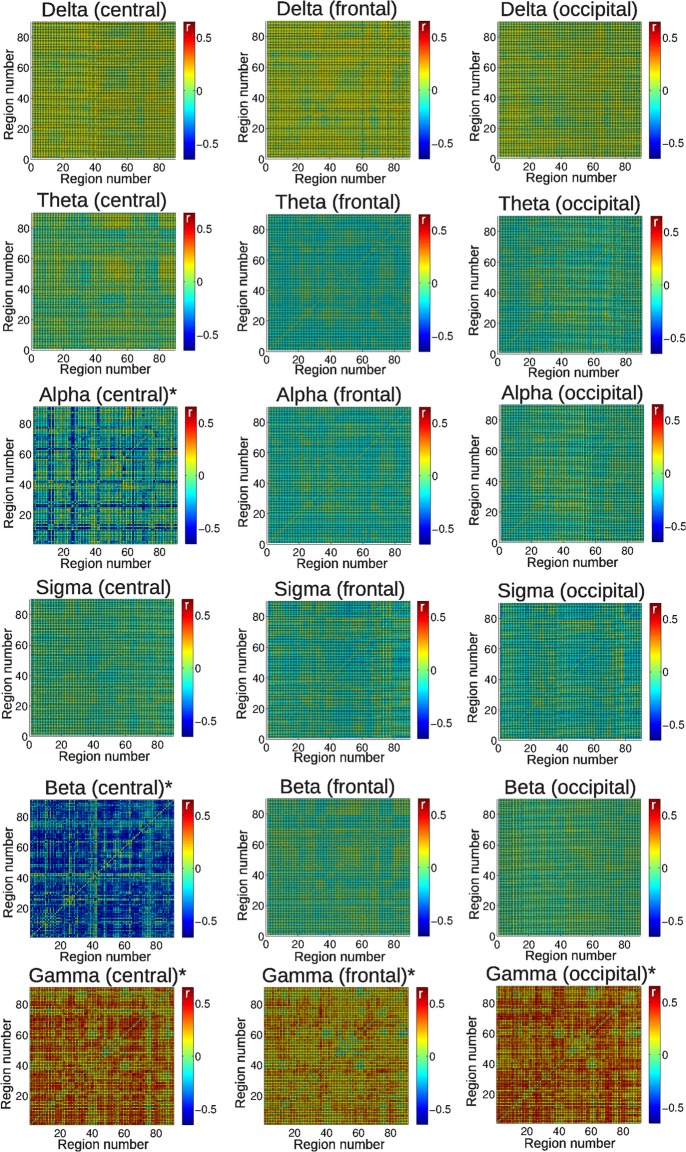
**Mean correlation between BOLD functional connectivity and EEG power fluctuations for all frequency bands and scalp regions under study**. Results are for the wake group of subjects. ^*^indicates the frequency bands and scalp regions for which significant correlations were found (see Figure 4). For the AAL regions associated with region numbers, see Table 1.

**Figure A2:**
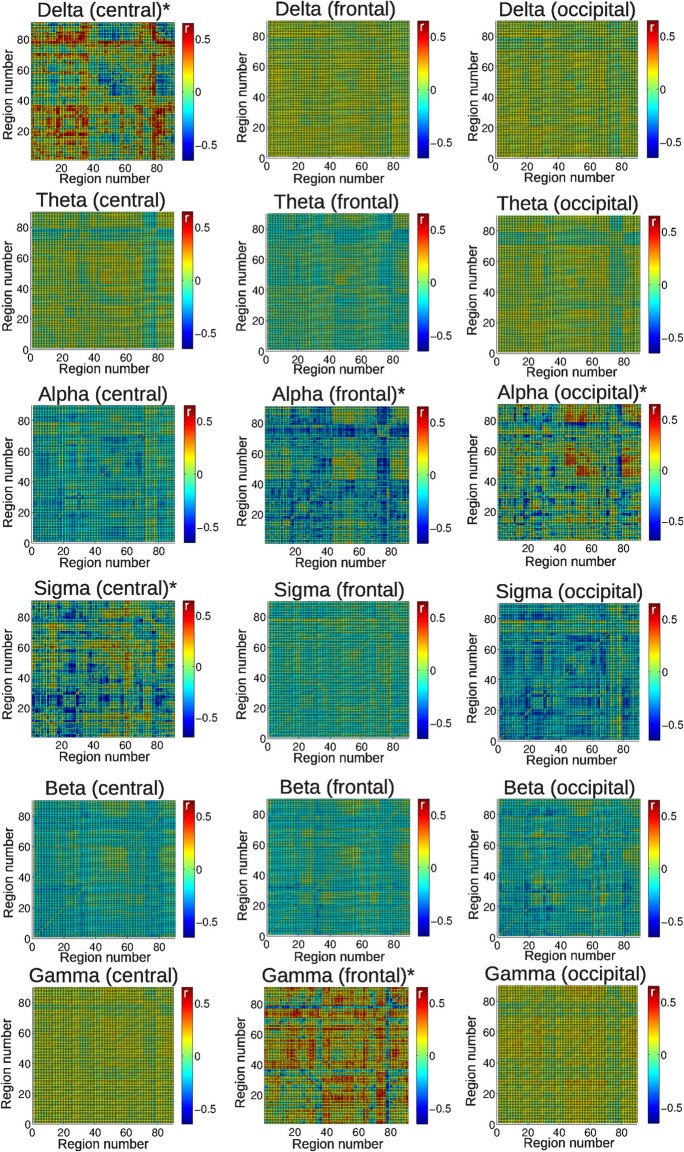
**Mean correlation between BOLD functional connectivity and EEG power fluctuations for all frequency bands and scalp regions under study**. Results are for the wake group of subjects. ^*^indicates the frequency bands and scalp regions for which significant correlations were found (see Figure 5). For the AAL regions associated with region numbers, see Table 1.

## Appendix

In Figures A1 and A2 all the mean correlation matrices (for both groups, all frequencies and scalp locations) are reproduced for comparison purposes. Those matrices including significant correlations (also reproduced in Figures 4 and 5) are marked with an asterisk.
